# The lone lifeline—a rare case report of a single right coronary circulation with hypoplastic left coronary system

**DOI:** 10.1093/ehjcr/ytag705

**Published:** 2026-09-19

**Authors:** Ani Abhishek Sharma, Deepanjan Bhattacharya, Sanjeev S Mukherjee, Saurav Banerjee, Kalyan Kumar Saha

**Affiliations:** Department of Cardiology, All India Institute of Medical Sciences, NH-34, Basantapur, Saguna, Kalyani, West Bengal 741245, India; Department of Cardiology, All India Institute of Medical Sciences, NH-34, Basantapur, Saguna, Kalyani, West Bengal 741245, India; Department of Cardiology, All India Institute of Medical Sciences, NH-34, Basantapur, Saguna, Kalyani, West Bengal 741245, India; Department of Cardiology, All India Institute of Medical Sciences, NH-34, Basantapur, Saguna, Kalyani, West Bengal 741245, India; Department of Cardiology, All India Institute of Medical Sciences, NH-34, Basantapur, Saguna, Kalyani, West Bengal 741245, India

**Keywords:** Single coronary artery, Hypoplastic coronary artery disease, Congenital coronary anomaly, Case report

## Abstract

**Background:**

Single coronary artery is an exceptionally rare congenital anomaly, occurring in ∼0.02% of patients undergoing coronary angiography. Hypoplastic coronary artery disease represents another uncommon congenital abnormality characterized by underdevelopment of epicardial coronary arteries. The coexistence of these anomalies resulting in a functionally single coronary circulation is extremely rare and has important diagnostic and clinical implications.

**Case summary:**

A woman in her late 60s presented with atypical chest pain. Electrocardiography and transthoracic echocardiography demonstrated normal findings with preserved biventricular function. Coronary angiography revealed an atretic left coronary ostium with retrograde filling of the left coronary system from the right coronary artery. Computed tomography coronary angiography confirmed a dominant right coronary artery supplying the entire myocardium with a hypoplastic left coronary system. The left anterior descending artery was opacified via collateralization through Vieussens’ arterial ring. No obstructive coronary disease was identified. The patient was managed conservatively and remained clinically stable on follow-up.

**Discussion:**

Rare coronary artery anomalies may present with non-specific symptoms and require multimodality imaging for definitive diagnosis. Early recognition is crucial for appropriate risk stratification, management, and surveillance.

Learning points

- Single coronary artery and hypoplastic coronary artery disease may present incidentally, with atypical symptoms, or rarely with life-threatening events.
- Clinical manifestations depend on the coronary course, myocardial territory supplied, and adequacy of collateral circulation.

## Introduction

Congenital coronary artery anomalies are rare but clinically relevant entities, with a reported prevalence of less than 1% in the general population. Single coronary artery (SCA), in which the entire coronary circulation arises from a single ostium, is an exceptionally uncommon anomaly, identified in ∼0.02% of patients undergoing coronary angiography. Another rare congenital entity is hypoplastic coronary artery disease (HCAD), characterized by underdevelopment of one or more epicardial coronary arteries, which may result in myocardial ischaemia, arrhythmias, or sudden cardiac death despite the absence of atherosclerotic disease. Although many coronary anomalies remain asymptomatic, clinical manifestations depend largely on the arterial course and the adequacy of collateral circulation. With the increasing use of invasive coronary angiography and computed tomography coronary angiography, recognition and anatomical delineation of these anomalies have improved. We report a rare case of a functionally single coronary circulation arising from the right coronary sinus with features suggestive of HCAD and retrograde filling of the left coronary system via Vieussens’ arterial ring, underscoring the diagnostic and clinical importance of this collateral pathway.

## Summary figure

**Figure:**
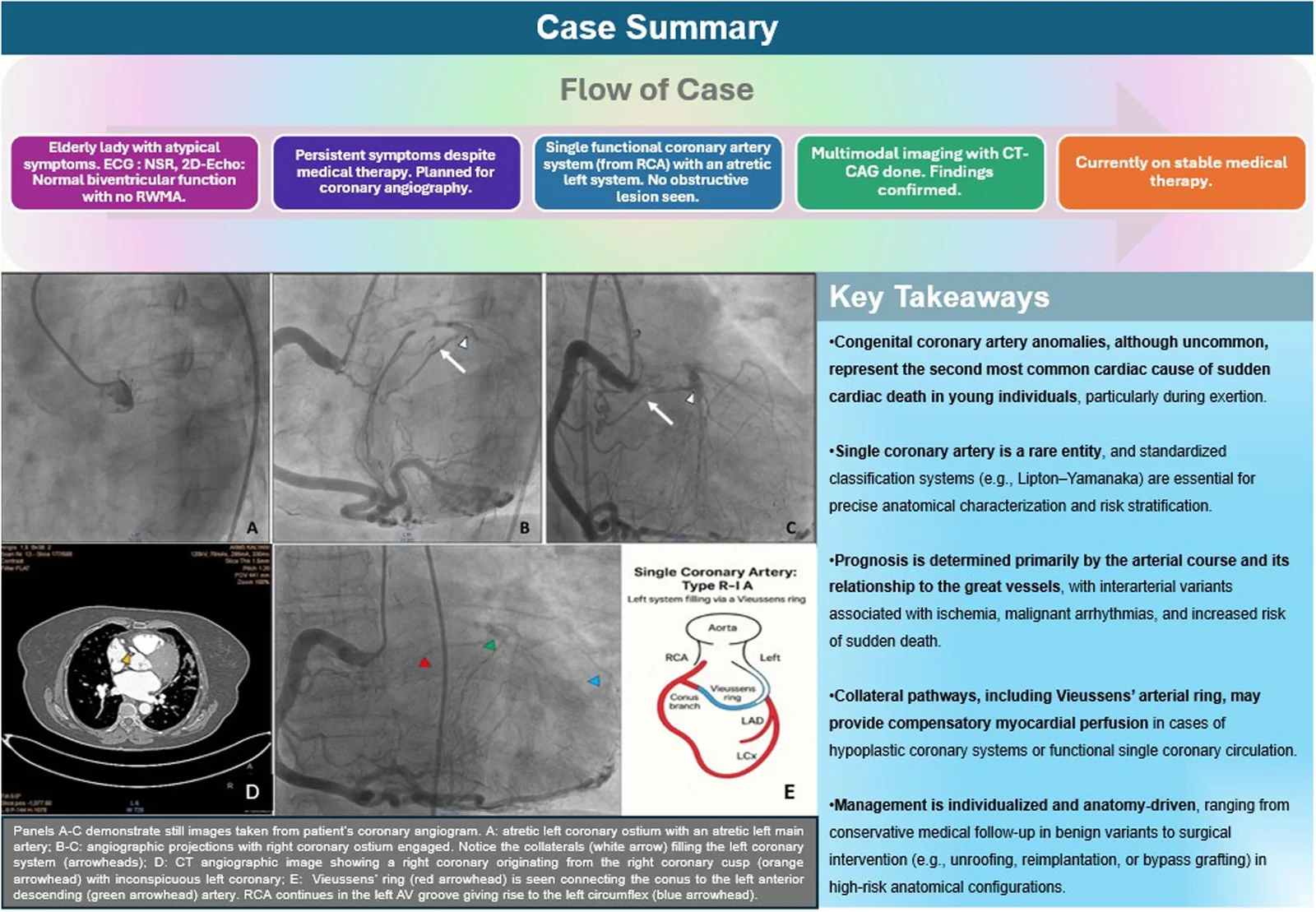


## Case presentation

An elderly lady, in her late 60s, presented to the outpatient department with atypical chest pain. She had no previously known comorbidities. General physical examination and systemic examination were unremarkable. ECG showed normal sinus rhythm, and transthoracic echocardiography was indicative of normal biventricular function with no regional wall motion abnormality or any significant valvular lesion. Routine blood investigations were within normal limits. Medical management was tried with a beta-blocker, nitrates, and a proton-pump inhibitor to no avail, and the patient was posted for a stress (exercise) test, which she could not complete on account of dyspnoea during stage 2 of the modified Bruce protocol. Coronary angiography was thus planned to rule out coronary artery disease. Coronary angiogram (*Figure 1*) revealed a single functional coronary circulation with an atretic left main trunk and retrograde opacification of the entire left coronary system from the right coronary artery (RCA). The patient was subsequently taken up for CT coronary angiography for anatomical delineation, which then confirmed the findings (*Figure 1F*). No obstructive lesion was seen on either. The patient was continued on medical management and is currently doing well on follow-up.

**Figure 1:**
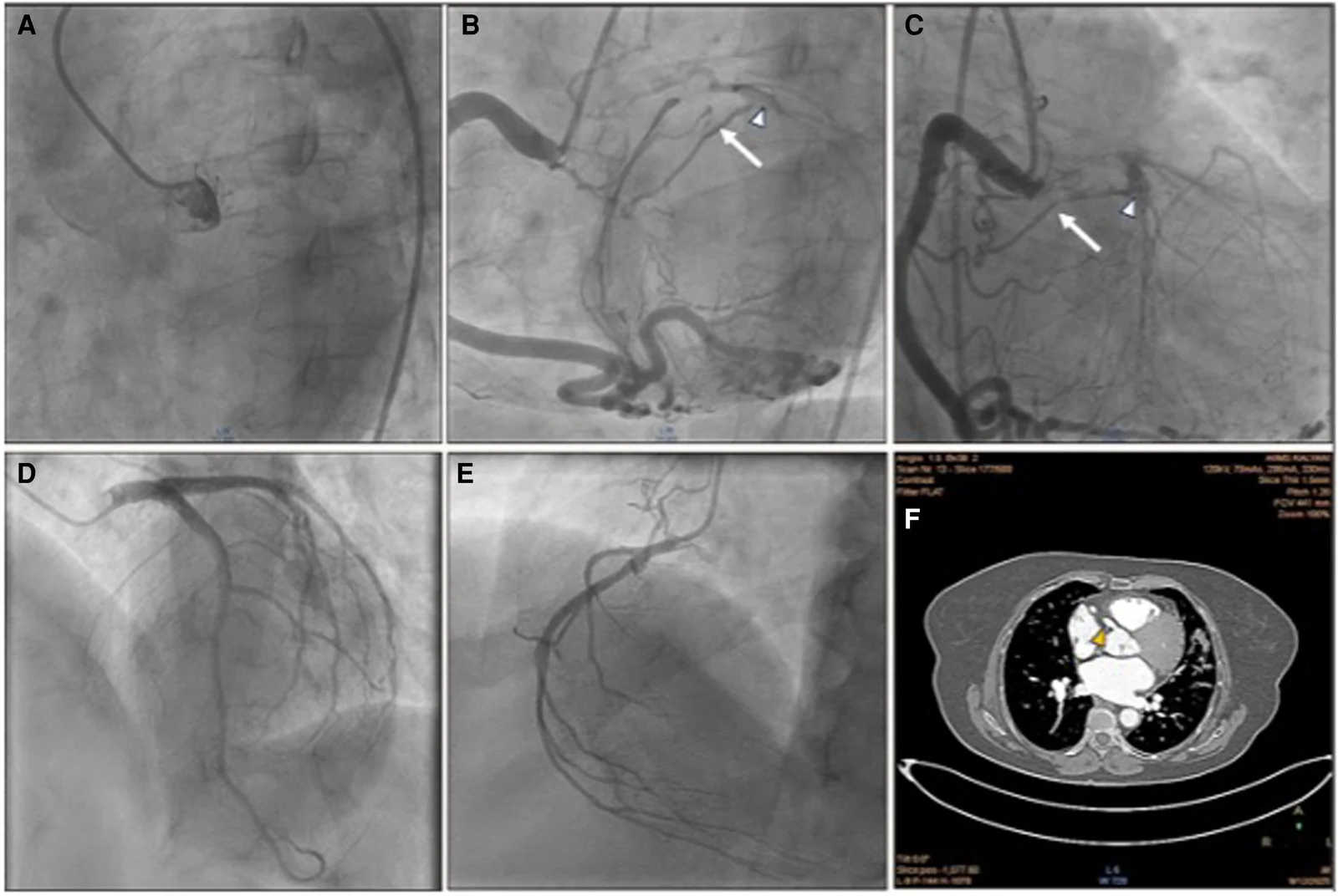
Evaluation of the patient. Panels (*A–C*) demonstrate still images taken from patient’s coronary angiogram. (*A*) Atretic left coronary ostium with an atretic left main artery (compare with panel *D*, which demonstrates a normal angiographic view of the left system); (*B* and *C*) angiographic projections with right coronary ostium engaged. Notice the collaterals (white arrow) filling the left coronary system (arrowheads). Compare the calibre of the vessel with panel *E*. (*D*) Angiographic projection showing a normal left coronary system. (*E*) Angiographic projection showing a normal right coronary system. (*F*) CT angiographic image showing a right coronary originating from the right coronary cusp (orange arrowhead) with inconspicuous left coronary.

## Discussion

Coronary artery anomalies form one of the more confusing and often neglected sects of cardiology. By convention, a variant is defined as an *anomaly* if incidence in the general population is <1%.^1^ Single coronary artery is found in ∼0.02% of the general population,^2^ of which a single coronary artery arising from the right ostium represents 2%–4%.^3^ Symptoms range from asymptomatic to stable angina to sudden death, with coronary artery anomalies frequently being heralded as the second most common cardiac cause of sudden death in the young, occurring often after strenuous exercise.^4^ Frequently, SCAs coexist with other congenital conditions such as a bicuspid aortic valve, pulmonary atresia, or transposition of great vessels. The quantum of symptoms depends on the arterial course—hence, SCAs were initially classified by Lipton *et al*.^5^ (and later modified by Yamanaka and Hobbs^3^) based on the origin, arterial course, and relationship to great vessels (*Figure 2*). Anterior (A), posterior (P), subpulmonic (S), and combined (C) courses are generally considered benign (do not impair perfusion), while arterial course B (interarterial) has been associated with exercise-induced arrhythmias, syncope, or even sudden cardiac death in young or athletic patients and often requires surgical intervention—unroofing of intramural segment, coronary re-implantation, or coronary artery bypass grafting (depending on the anatomy).

**Figure 2:**
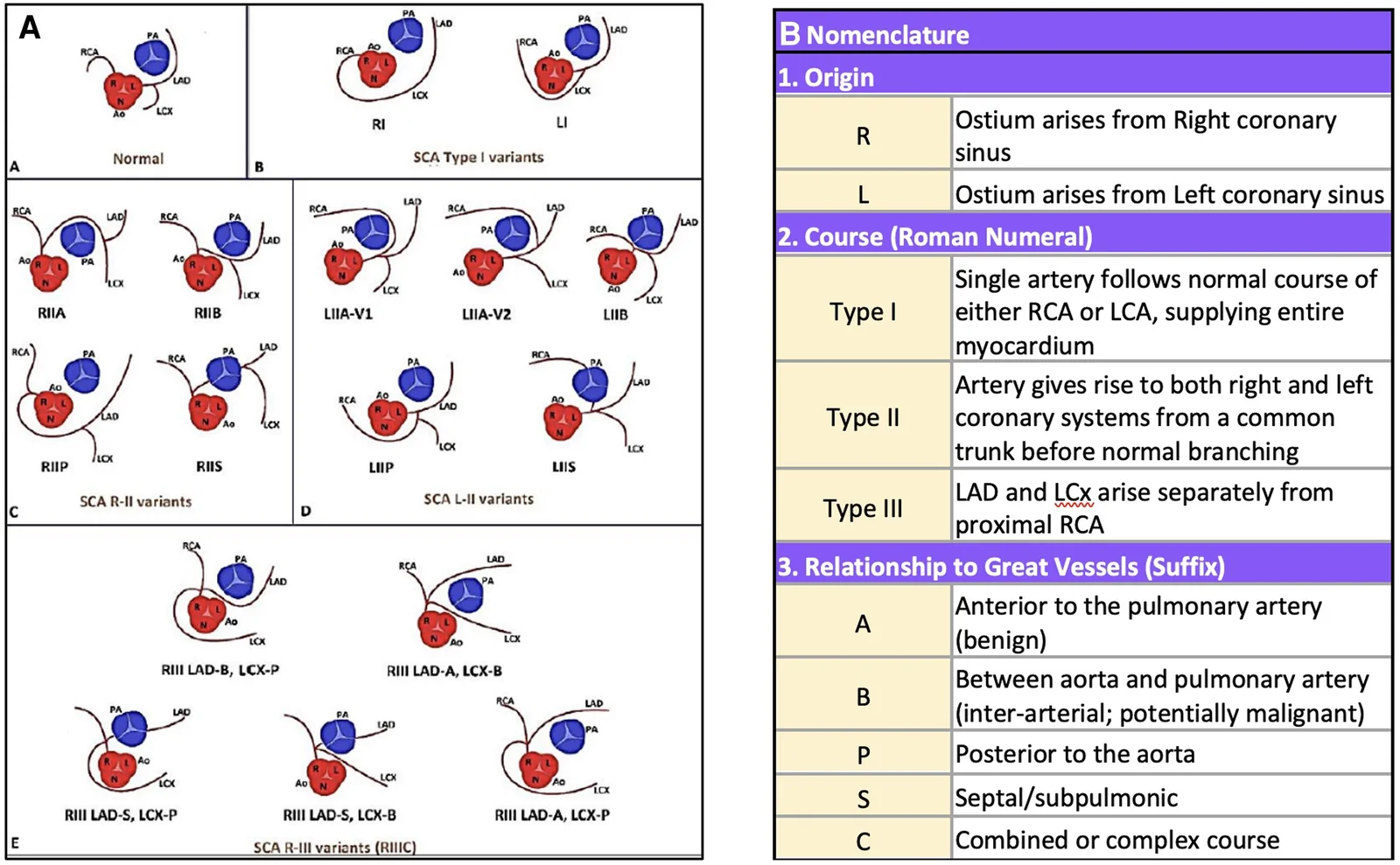
The Lipton–Yamanaka classification for single coronary artery. (*A*) The various arterial routes; (B) represents the nomenclature as per the arterial course.

Our case represents a single coronary artery of type R-I-A with the left system filling from the right system through Vieussens’ ring (*Figure 3*). Vieussens’ ring is a normal coronary arterial anastomosis that connects the conus branch of the RCA to the proximal left anterior descending artery (or its septal branches), which often becomes prominent during chronic occlusions and often plays an important role in planning of interventions in chronic total occlusions. Conventional coronary angiography in conjunction with CT coronary angiography confirmed the anatomy, also revealing a hypoplastic left coronary system. Hypoplastic coronary artery disease was first described in the 1970s and is a rather rare entity with <50 cases reported worldwide.^6^ Symptoms overlap with those of SCA, and management options remain largely the same. Across the reported cases, hypoplastic right coronary and left circumflex are the most commonly implicated arteries,^6,7^ which makes our case even more curious. We hypothesize our patient to be a case of HCAD with compensatory enlargement of the right coronary system with recruitment of collateral systems such as the Vieussens’ ring culminating in a single functional coronary system. Clinical suspicion, though not pragmatic in all cases given the rarity of the entity, precludes missed diagnoses and may save a life in case of a malignant arterial course.

**Figure 3:**
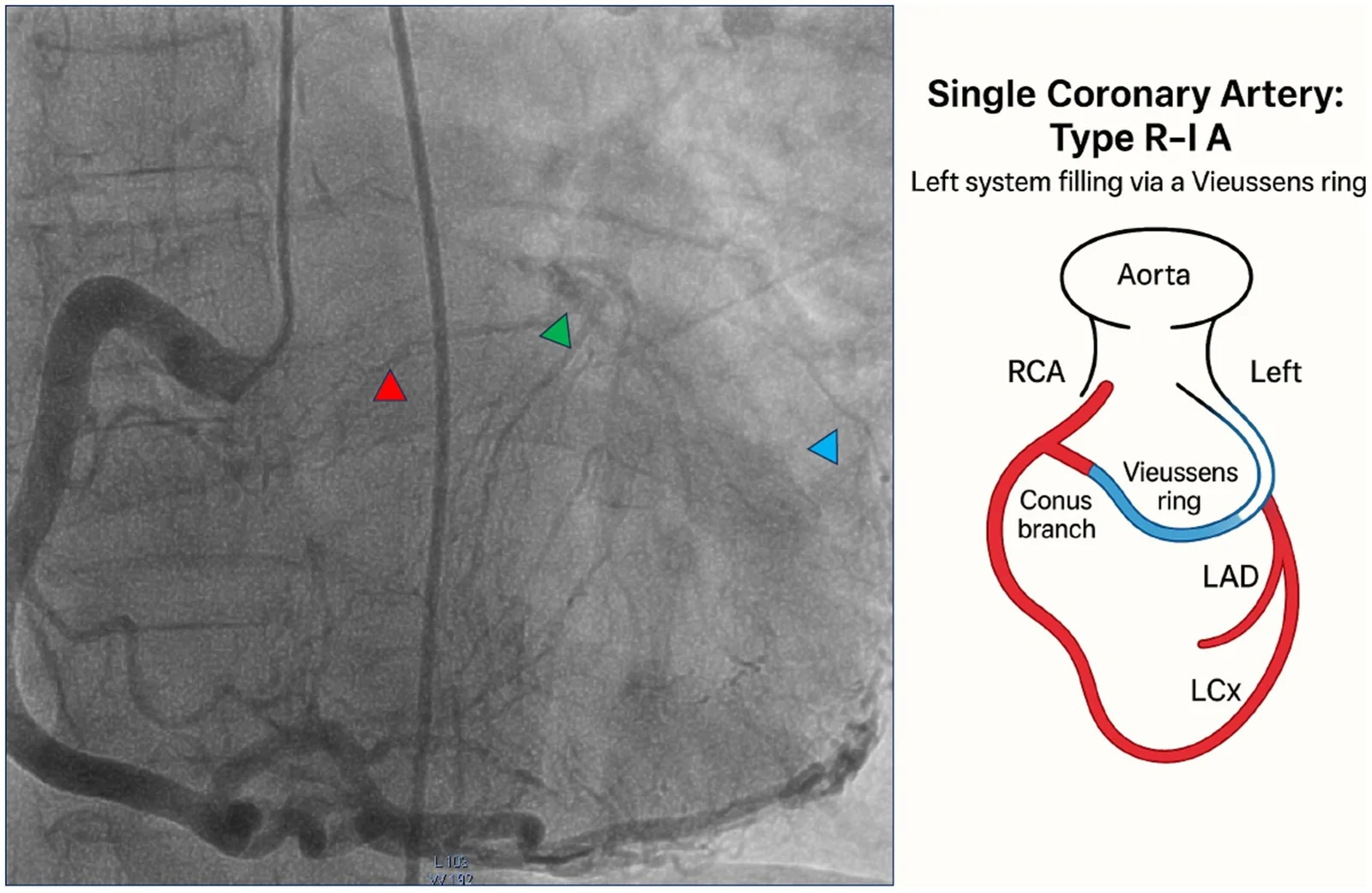
Angiographic projection of the patient (right anterior oblique cranial). Vieussens’ ring (red arrowhead) is seen connecting the conus to the left anterior descending (green arrowhead) artery. Right coronary artery continues in the left atrioventricular groove, giving rise to the left circumflex (blue arrowhead).

## Data Availability

All data underlying this article are available within the article. The original patient-level DICOM imaging data, including coronary angiography and CT scan datasets, are not publicly available to preserve patient confidentiality.

## Lead author biography

Dr. Ani Abhishek Sharma is a Doctorate of Medicine (DM) resident in Cardiology at the All India Institute of Medical Sciences (AIIMS), Kalyani, India. He completed his MBBS from Government Medical College, Kota, followed by MD and DNB in General Medicine. His academic interests include coronary artery disease, cardiovascular imaging, structural heart disease, and heart failure. He is actively involved in clinical research with a focus on echocardiographic strain imaging and cardiac MRI and is committed to advancing evidence-based cardiovascular care through clinical practice, research, and medical education.

## Notes

**Consent:** Written informed consent was obtained from the patient for publication of this case report and any accompanying images. A copy of the written consent is available for review on request.
